# *Escherichia coli* and *Salmonella* spp. isolated from Australian meat chickens remain susceptible to critically important antimicrobial agents

**DOI:** 10.1371/journal.pone.0224281

**Published:** 2019-10-23

**Authors:** Sam Abraham, Mark O’Dea, Shafi Sahibzada, Kylie Hewson, Anthony Pavic, Tania Veltman, Rebecca Abraham, Taha Harris, Darren J. Trott, David Jordan

**Affiliations:** 1 Antimicrobial Resistance and Infectious Disease Laboratory, Murdoch University, Murdoch, Western Australia, Australia; 2 Australian Chicken Meat Federation, Sydney, New South Wales, Australia; 3 Birling Avian Laboratories, Bringelly, New South Wales, Australia; 4 Australian Centre for Antimicrobial Resistance Ecology, School of Animal and Veterinary Sciences, The University of Adelaide, Roseworthy, South Australia, Australia; 5 New South Wales Department of Primary Industries, Wollongbar, New South Wales, Australia; Australian National University, AUSTRALIA

## Abstract

The World Health Organisation has defined “highest priority critically important antimicrobials” (CIAs) as those requiring the greatest control during food production. Evidence demonstrating that restricted antimicrobial usage prevents the emergence of resistance to CIA’s amongst pathogenic and commensal organisms on a production system-wide scale would strengthen international efforts to control antimicrobial resistance (AMR). Therefore, in a designed survey of all major chicken-meat producers in Australia, we investigated the phenotypic AMR of *E*. *coli* (n = 206) and *Salmonella* (n = 53) from caecal samples of chickens at slaughter (n = 200). A large proportion of *E*. *coli* isolates (63.1%) were susceptible to all tested antimicrobials. With regards to CIA resistance, only two *E*.*coli* isolates demonstrated resistance to fluoroquinolones, attributed to mutations in the quinolone resistance-determining regions of *gyrA*. Antimicrobial resistance was observed for trimethoprim/sulfamethoxazole (8.7%), streptomycin (9.7%), ampicillin (14.1%), tetracycline (19.4%) and cefoxitin (0.5%). All *Salmonella* isolates were susceptible to ceftiofur, chloramphenicol, ciprofloxacin, colistin, florfenicol, gentamicin and tetracycline. A low frequency of *Salmonella* isolates exhibited resistance to streptomycin (1.9%), ampicillin (3.8%), and cefoxitin (11.3%). AMR was only observed among *Salmonella* Sofia serovars. None of the *Salmonella* isolates exhibited a multi-class-resistant phenotype. Whole genome sequencing did not identify any known resistance mechanisms for the *Salmonella* isolates demonstrating resistance to cefoxitin. The results provide strong evidence that resistance to highest priority CIA’s is absent in commensal *E*. *coli* and *Salmonella* isolated from Australian meat chickens, and demonstrates low levels of resistance to compounds with less critical ratings such as cefoxitin, trimethoprim/sulfamethoxazole, and tetracycline. Apart from regulated exclusion of CIAs from most aspects of livestock production, vaccination against key bacterial pathogens and stringent biosecurity are likely to have contributed to the favorable AMR status of the Australian chicken meat industry. Nevertheless, industry and government need to proactively monitor AMR and antimicrobial stewardship practices to ensure the long-term protection of both animal and human health.

## Introduction

Pathogenic Gram-negative bacteria resistant to highest priority critically important antimicrobials (CIAs) including extended-spectrum cephalosporins (ESCs), fluoroquinolones, carbapenems, and colistin, are a significant public health threat due to limited therapeutic options for treatment [1]. CIA-resistant bacteria have been detected in food-producing animals with the number of reports identifying CIA-resistant bacteria in animals markedly increasing in recent years [2, 3]. These resistant bacteria present a possible risk of direct transmission via cross-infection and colonisation of humans or indirect transfer of associated mobile genetic elements to potentially pathogenic organisms of the human gastrointestinal tract via the food chain [4].

Globally, resistance to fluroquinolones is common among *E*. *coli* and *Salmonella* isolated during broiler-meat production in Asia, Northern America and Europe while resistance to third-generation cephalosporins and colistin is also widespread internationally being common in some countries. In a recent review [5] the rates of resistance in *E*. *coli* isolates from healthy broilers were compared from available studies in a number of countries (USA, Brazil, China, Poland, United Kingdom, Germany, France and Spain). For ciprofloxacin, all countries except the USA had a median (across all studies performed in that country) percent of isolates resistant exceeding 20%, and typically in the range 40% to 60%. For third generation cephalosporins (ceftiofur and cefotaxime), all countries except the United Kingdom, showed conclusive evidence of resistance being present (median resistance exceeding 5% of isolates, with some countries as high as 50%). For *Salmonella* spp, isolated from broiler carcases the rates of resistance across 19 European Union member states is very high for ciprofloxacin (64.7%) with only three member states having zero detections [6]. With reference to colistin resistance in *E*. *coli* and *Salmonella* from poultry a recent international review shows resistance as being widespread in both developed and developing countries, often exceeding 5% of isolates [7].

Recent studies suggest that the ecology of antimicrobial resistance among key Gram-negative bacteria isolated from Australian livestock, such as *E*. *coli* and *Salmonella*, may be different to that of many other countries [8–10]. These differences include no detected resistance to colistin and carbapenems, and relatively low resistance (suggesting either very recent emergence or negligible co-selection) to fluoroquinolones (0–1%) and ESCs (0–3%) among commensal *E*. *coli*, pathogenic *E*. *coli* and *Salmonella* isolated from pigs, cattle and sheep [11, 12]. Additionally, a new clade of ESC-resistant *Salmonella* serovar Typhimurium has recently been identified in a cluster of sporadic clinical cases of salmonellosis in both humans and dairy cattle in the state of Victoria [13]. The comparatively low levels of CIA resistance in Australian food-producing animals are attributed to Australia’s geographic isolation, strong regulatory constraints on the use of CIAs, such as ESCs, fluoroquinolones and colistin, and strict quarantine on the importation of live animals and fresh meat products.

The market share of chicken meat amongst other meats in Australia has trebled in recent decades [14] and it is currently the meat with the highest per capita consumption [15]. This high level of consumption combined with limited scrutiny of the AMR status of meat chickens has meant this class of livestock is underrepresented in the limited studies of AMR in Australian food-producing animals. The only previous national survey of AMR conducted among healthy Australian meat chickens was undertaken in 2003/2004 [16], and Australia currently does not have an ongoing national AMR surveillance programme focused on healthy livestock. However, recent cross-sectional surveys on poultry-derived retail products have returned mixed results, with *E coli* isolation rates as low as 0.8% [17–19]. In addition, mixed resistance profiles, including the detection of CIAs (fluoroquinolones) at low frequency, and diverse phylogenetic groups have led authors to hypothesise that contamination of retail poultry meat in Australia is potentially occurring at multiple stages in the food chain and is not necessarily due to contamination present prior to slaughter [17–19]. To assess this, a sampling programme conducted on poultry at the point of slaughter is required.

In the current study, we assessed the frequency of AMR among *E*. *coli* and *Salmonella* isolated from meat chickens representing all the major producers in Australia using a structured, epidemiologically-based approach to sampling combined with the adoption of whole genome sequencing to comprehensively characterise isolates from both AMR and ecological perspectives. It was hypothesised that *E*. *coli* and *Salmonella* isolated from meat chickens in Australia would be fully susceptible to highest priority CIAs, and, that resistance to antimicrobial agents of lesser importance to human health was likely to be low, due to the conservative use of antimicrobials in the Australian meat chicken industry.

## Materials and methods

The basis for the sampling methods adopted in this study were the National Antimicrobial Resistance Monitoring System (NARMS) recommendations for surveillance in the USA [20]. Study design and sampling method is explained in detail in O’Dea, Sahibzada [21] and aimed to procure a collection of isolates reflecting the population of *E*. *coli* entering the food-chain in the gut of meat chickens. In summary, a total of 200 pooled caecal samples, each consisting of five composite caeca, were acquired, processed and assayed. Caeca were collected between June and November 2016, from twenty poultry abattoir plants owned by seven commercial companies that process approximately 11 million chickens per week, representing 95% of Australian chicken meat production.

Following transport in chilled containers (<8 °C) to the laboratory, caecal samples were homogenised in buffered peptone water within 24h post collection from abattoirs, plated onto *E*. *coli* -specific chromogenic agar (Coli ID^™^, BioMerieux, France) and incubated aerobically at 37°C for 18h. One single, presumptive *E*. *coli* colony per plate was selected and subcultured for purity, with identity confirmed by indole spot test and MALDI-TOF mass spectroscopy (Bruker Microflex). From a pure subculture on nutrient agar (Accumedia, USA), bacteria were harvested for storage at -80°C on cryo-beads (Cryobank, Mast Diagnostics). *Salmonella* was isolated using a fully validated modification of the AS 5013.10–2009 method for *Salmonella* spp. which is similar to the International Organization for Standardization (6579–1) [22] using Rappaport-Vassiliadis (RV) and Muller-Kauffmann (MK) media (Edwards, Australia). Isolates were cultured onto differential and selective media, XLD, and Hektone (BioMerieux, Australia). Presumptive confirmation was performed using SMID2 (BioMerieux, France) and species confirmation was performed on all isolates using MALDI-TOF.

### Salmonella serotyping

Presumptively identified *Salmonella* were serologically confirmed with Poly O and H antisera (Prolab Diagnostics, Canada) after subculture onto two slopes of nutrient agar (Accumedia, USA) then employing the slide agglutination technique.

### Antimicrobial Susceptibility Testing

Antimicrobial susceptibility testing on each of the *E*. *coli* and *Salmonella* spp. isolates recovered from cryo-beads was performed by the broth microdilution MIC method using the Sensititre system and a CMV3AGNF Sensititre^®^ NARMS panel (Trek Diagnostics, Thermofisher Scientific). The MIC results were captured using Vision System (Trek Diagnostics, Thermo Fisher Scientific) and results interpreted and verified independently by two laboratory scientists. The isolates were tested against the following 13 different antimicrobials: amoxicillin-clavulanate, ampicillin, cefoxitin, ceftiofur, chloramphenicol, ciprofloxacin, colistin, ceftriaxone, florfenicol, gentamicin, streptomycin, tetracycline, and trimethoprim/sulfamethoxazole. MICs were interpreted using epidemiologic cut-off values (ECOFFs) following the guidelines of the European Committee on Antimicrobial Susceptibility Testing [23]. Where no EUCAST ECOFF interpretative criteria were available, provisional breakpoints were determined using ECOFF finder [24]. Quality control was performed using *E*. *coli* ATCC25922 throughout the study period.

Non-wild type isolates (defined as having MICs above the ECOFF) have been shown to contain acquired resistance mechanisms in their genome, even though they may have MICs below the defined CLSI clinical breakpoints [25]. Therefore, for more simplistic determination of individual and multi-class resistance profiles, we refer to isolates exceeding the antimicrobial ECOFF as “resistant”. Multi-class resistant (MCR) isolates are therefore defined as having MICs above the ECOFF for one or more antimicrobial agents in three or more antimicrobial classes. This enables comparison with AMR surveillance systems such as DANMAP (https://www.danmap.org) and is more useful for assessing the recent emergence of resistance to multiple classes, particularly in populations expected to have low levels of resistance.

### Whole genome sequencing

Whole genome sequencing was performed on isolates with MICs above the ECOFF for any highest priority CIA included in the test panel (n = 8). DNA was extracted using MagMAX Multi-sample DNA extraction kit (Thermo Fisher Scientific) as per the manufacturer’s instructions and the DNA library was prepared using Illumina Nextera XT Library Preparation kit (Illumina) with an extended tagmentation time of seven minutes. Genomic data was analysed as previously described [26]. The Nullarbor pipeline v1.01 (https://github.com/tseemann/nullarbor) was used to assemble the eight Illumina sequenced strains. The resulting FASTA files were analysed using the ResFinder, VirulenceFinder and PlasmidFinder functions of the Centre for Genomic Epidemiology database (http://www.genomicepidemiology.org/). All sequence read data generated in this study was deposited in the NCBI Sequence Read Archive under accession number PRJNA573547.

### Statistical analysis

Confidence intervals of proportions were calculated using exact binomial confidence intervals using the Clopper-Pearson method. Significance of differences between enterprises in the proportion of isolates resistant to at least one antimicrobial (judged as P < 0.05) were assessed using Fischer’s Exact Test. All analysis was performed in Stata version 15.1 (StataCorp LLC, College Station, Texas USA, www.stata.com).

## Results

*E*. *coli* was isolated from all 200 pooled caecal samples. A total of 206 *E*. *coli* isolates were chosen from the 200 pooled cultures based on the colony morphology. *Salmonella* spp. was recovered from 53 pooled samples (26.5%) with twelve different serotypes. The most frequent serovar was *Salmonella enterica salamae* Sofia (34.0%), followed by Serovars of *Salmonella enterica enterica* Abortusovis (15.1%), Adelaide (15.1%), and Typhimurium (7.6%). All serotypes detected in this study are outlined in Table 1.

**Table 1:** Distribution of *Salmonella* serotypes isolated from Australian meat chickens.

Serotype	Frequency	Percentage
Sofia	18	34.0
Abortusovis	8	15.1
Adelaide	8	15.1
Typhimurium	6	11.3
Virchow	4	7.6
Mbandaka	3	5.7
Infantis	1	1.9
Muenchen	1	1.9
Orion	1	1.9
Saintpaul	1	1.9
Senftenberg	1	1.9
Zanzibar	1	1.9

### Antimicrobial resistance among *E*. *coli*

None of the *E*. *coli* isolates exhibited resistance to amoxicillin-clavulanate, ceftiofur, chloramphenicol, colistin, florfenicol or gentamicin. Only two isolates demonstrated MICs above the ECOFF for CIAs. Fluoroquinolones resistance was observed in these two isolates with ciprofloxacin; MICs of 0.06 mg/L,however these MICs were below the CLSI clinical resistance breakpoints. Antimicrobial resistance was also observed for cefoxitin (0.05%), trimethoprim/sulfamethoxazole (8.7%), streptomycin (9.7%), ampicillin (14.1%), and tetracycline (19.4%) (Table 2]. There were no significant differences between enterprises in the proportion of isolates resistant to at least one antimicrobial.

**Table 2:** Distribution (percent of isolates) of minimum inhibitory concentrations (mg/L) for commensal *Escherichia coli* (n = 206) isolated from Australian meat chickens at slaughter.

Antimicrobial	Class	0.016	0.03	0.06	0.13	0.25	0.5	1	2	4	8	16	32	64	128	256	Percent non-wildtype (95% CI)
Amoxicillin-clavulanate	bla-i							5.3	39.3	42.7	12.6						0 (0–1.8)
Ampicillin	bla							19.9	44.2	21.4	0.5			14.1			14.1 (9.6–19.0)
Cefoxitin	c2g								4.9	70.4	24.3	0.5					0.5 (0–2.7)
Ceftiofur *	c3g				1.5	45.1	52.4	1									0 (0–1.8)
Ceftriaxone *	c3g					100											0 (0–1.8)
Chloramphenicol	phe								4.4	43.7	51.9						0 (0–1.8)
Ciprofloxacin *	qui	95.1	3.9		0.5	0.5											1 (0.1–3.5)
Colistin *	pol				21.8	73.3	3.4	1.5									0 (0–1.8)
Florfenicol	phe									9.7	76.2	14.1					0 (0–1.8)
Gentamicin	ami					5.8	79.1	15									0 (0–1.8)
Streptomycin	ami								1	50	36.9	2.4	4.9	2.4	2.4		9.7 (6–14.6)
Tetracycline	tet									80.6				19.4			19.4 (14.2–25.5)
Trimethoprim/sulf	fpi				87.9	1.5	1.5	0.5			8.7						8.7 (5.3–13.5)

The shaded areas indicate the range of dilutions tested for each antimicrobial. ECOFF values are shown with vertical bars. ami = aminoglycosides, bla = beta lactams, bla-i = beta lactams/inhibitor, c2g = 2^nd^ generation cephalosporins, c3g = 3^st^ generation cephalosporin, fpi = folate pathway inhibitors, phe = phenicols, pol = polymixins, qui = quinolones, tet = tetracycline

*—Critically important antimicrobial

A total of 15 resistance profiles were identified among the 206 *E*. *coli* isolates. However, 66.5% of the isolates were not resistant to any of the tested antimicrobials. Only 5.4% of the *E*. *coli* isolates were classified as MCR, with the most common MCR profile of β-lactams, folate pathway inhibitors and tetracycline resistance shared by six isolates (Table 3). One isolate demonstrated resistance to four classes (ami_bla_fpi_tet) of antimicrobials and another to five classes [ami_bla_fpi_qui_tet] (Table 3), none of which were CIAs.

**Table 3:** Antimicrobial resistance profiles of *Escherichia coli* isolates from Australian poultry based on ECOFFs (n = 206).

No. of classes: phenotype	Frequency	Percentage
0: nil	137	66.5
1: ami	10	4.9
1: bla	11	5.3
1: fpi	4	1.9
1: tet	17	8.3
2: ami_qui	1	0.5
2: ami_tet	4	1.9
2: bla_c2g	1	0.5
2: bla_tet	6	2.9
2: fpi_tet	4	1.9
3: ami_bla_fpi	2	1.0
3: ami_bla_tet	1	0.5
3: bla_fpi_tet	6	2.9
4: ami_bla_fpi_tet	1	0.5
5: ami_bla_fpi_qui_tet	1	0.5

ami = aminoglycosides, bla = beta lactams, c1g = 2^nd^ generation cephalosporins, fpi = folate pathway inhibitors, qui = quinolones, tet = tetracycline

### Antimicrobial resistance among *Salmonella spp*.

None of the *Salmonella* isolates was resistant to ceftiofur, chloramphenicol, ciprofloxacin, colistin, florfenicol, gentamicin or tetracycline. A low frequency of *Salmonella* isolates exhibited resistance to streptomycin (1.9%), ampicillin (3.8%), and cefoxitin (11.3%) (Table 4). AMR was only observed among *Salmonella* Sofia serovar and all other serovars including *Salmonella* Typhimurium were susceptible to all tested antimicrobials. None of the *Salmonella* isolates exhibited an MCR phenotype. There was no significant difference between enterprises in the proportion of isolates expressing any form of resistance.

**Table 4:** Distribution (percent of isolates) of minimum inhibitory concentrations (mg/L) for *Salmonella* spp (n = 206) isolated from Australian meat chickens at slaughter.

Antimicrobial	Class	0.016	0.03	0.06	0.13	0.25	0.5	1	2	4	8	16	32	64	128	256	Percent non-wildtype (95% CI)
Amoxicillin-clavulanate	bla-i							77.4	17	1.9	1.9	1.9					3.8 (0.5–13)
Ampicillin	bla							67.9	28.3					3.8			3.8 (0.5–13)
Cefoxitin	c2g								34	41.5	13.2	11.3					11.3 (4.3–23)
Ceftiofur *	c3g					1.9	18.9	64.2	15.1								0 (0–6.7)
Ceftriaxone *	c3g					100											0 (0–6.7)
Chloramphenicol	phe									45.3	54.7						0 (0–6.7)
Ciprofloxacin *	qui	49.1	50.9														0 (0–6.7)
Colistin *	pol					9.4	60.4	30.2									0 (0–6.7)
Florfenicol	phe									24.5	71.7	3.8					0 (0–6.7)
Gentamicin	ami					66	34										0 (0–6.7)
Streptomycin	ami									20.8	60.4	17			1.9		1.9 (0–10.1)
Tetracycline	tet									100							0 (0–6.7)
Trimethoprim/sulfa	fpi				96.2	1.9					1.9						1.9 (0–10.1)

The shaded areas indicate the range of dilutions tested for each antimicrobial. ECOFF values are shown with vertical bars. ami = aminoglycosides, bla = beta lactams, bla-i = beta lactams/inhibitor, c2g = 2^nd^ generation cephalosporins, c3g = 3^st^ generation cephalosporin, fpi = folate pathway inhibitors, phe = phenicols, pol = polymixins, qui = quinolones, tet = tetracycline

*—Critically important antimicrobial

### Whole genome sequencing

Whole genome sequencing was performed on two *E*. *coli* isolates with elevated MICs (0.125 and 0.25 mg/L) above the ciprofloxacin ECOFF, identified as belonging to ST752 and ST4980, respectively. Both of these carried a single point mutation in the QRDR of *gyrA*, with the ST752 and ST4980 isolates each displaying amino acid mutations of Glu-475-Asp, and Ser-83-Leu and Asp-87-Gly, respectively (Table 5).

**Table 5:** MLST and profile of resistance genes identified in *Escherichia coli* isolates subjected to whole genome sequence analysis (n = 3).

Isolate ID	MLST	Resistance profile	Ciprofloxacin MIC (mg/L)	QRDR Mutations	Amino Acid Substitution
Chick_021	ST38	*bla*_TEM-1C_- *sul2*	0.015	Not detected	Not detected
Chick_133	ST752	*strA- strB*	0.25	GyrA	Ser (83)—Leu
				ParC	Glu (475)—Asp
Chick_202	ST4980	*bla*_TEM-1B_- *dfrA14- strA- strB- sul2- tetA*	0.12	GyrA	Asp (87)—Asn
				ParC	Glu (475)—Asp

All *Salmonella* isolates (Sofia) that had elevated MICs for cefoxitin (n = 6; 16 mg/L) were subjected to whole genome sequence analysis. Sequencing revealed all of these *Salmonella* isolates belonged to sequence type ST2116 (*Salmonella* Sofia), with no known antimicrobial resistance genes detected for any antimicrobials.

## Discussion

In this study, we investigated the antimicrobial resistance characteristics of *E*. *coli* and *Salmonella* isolated from meat chickens in Australia. The results strongly support our hypothesis that highest priority CIA resistance is absent in commensal *E*. *coli* and *Salmonella* isolated from Australian meat chickens. In addition, the current study also demonstrated low levels of resistance to antimicrobials with less critical ratings such as cefoxitin, trimethoprim/sulfamethoxazole, and tetracycline among both *E*. *coli* and *Salmonella* spp. isolated from Australian meat chickens when compared to other countries where resistance in these organisms has also been well studied [27, 28]. A MCR phenotype was observed only among a small number of *E*. *coli* isolates (5.4%), while none of the *Salmonella* isolates were identified as MCR. The findings are consistent with other recent studies demonstrating low levels of antimicrobial resistance among *E*. *coli* and *Salmonella* isolated from Australian food-producing animals [9, 12, 29], although higher levels of resistance to drugs with a lower importance rating was observed in a similar national AMR survey focused on healthy Australian pigs at slaughter [12].

The absence of resistance to ESCs amongst all the isolates in this study is noteworthy. Internationally, the drug ceftiofur has been widely used for several decades in commercial chicken flocks, while during that time registration and label constraints have precluded its use in Australian flocks [30]. In several countries where well developed surveillance systems are in place, the threat to public health arising from *Salmonella* spp. resistant to ESCs derived from animals is well established [27, 28, 31]. For example, Canada has experienced public health events related to chicken-meat consumption involving *Salmonella* Heidelberg resistant to ESCs, and a concomitant rise in commensal *E*. *coli* in retail chicken-meat with this same form of resistance [27, 32]. In the United States up to 10% of *Salmonella* spp. isolated from chicken caeca were found to be resistant to ESCs, and 15% were reported as MCR [28]. In the previous NARMS report, unlike the current study, a higher resistance level was also reported against tetracycline (37%) and streptomycin (30%) for non-typhoidal *Salmonella* isolated from chicken caeca in 2014.

A direct comparison between countries for resistance prevalence is complicated by heterogeneity between studies such as sampling source (retail food vs caecal samples), assays used for antimicrobial testing (broth vs agar dilution), and breakpoints (ECOFF vs CLSI clinical). However, trends in the pattern of resistance can be observed between surveys using similar sampling designs and interpretation tools. The present AMR survey in Australian meat chickens using caecal samples is comparable to the Canadian Integrated Program for Antimicrobial Resistance Surveillance (CIPARS) and NARMS in the United States due to similarity in the sampling and laboratory methodologies. A lower prevalence of resistance was observed against several antimicrobials tested when compared to the Canadian and the U.S. surveillance data. In comparison to CIPARS, the resistance among *E*. *coli* in the current study was markedly lower for all tested antimicrobials, for example ampicillin (14.1% vs 36.7%) and tetracycline (19.4% vs 48.3%) [27]. Using the same ECOFF values adopted in this study, the resistance level against tested antimicrobials was also comparatively lower than the reported frequency from *E*. *coli* isolated from chicken caecal samples in the USA where non-wild type was reported against gentamicin (53.6%), streptomycin (58.3%), and tetracycline (48.87%) [28]. Further comparisons using the present data should be made with caution since interpretations need to account for differences in methodology. For example, resistance to tetracycline in the present 2017 survey was lower (19.4% CI 14.2–25.5) than the 2004 survey of *E*. *coli* in Australian meat chickens (44.2% CI 38.2–50.4) [16]. However, the latter assessed sensitivity to antimicrobials using agar dilution assays, so it cannot be firmly concluded that the reduction over time in resistance is a true reduction due to environmental selection for fitness combined with a reduced selection for resistance in poultry production. A combination of factors is likely to have resulted in the low levels of resistance observed in this study.

All *E*. *coli* isolates had MICs below the ECOFF for all highest priority critically important antimicrobials in the test panel inclusive of ciprofloxacin, ceftriaxone, ceftiofur and colistin [1] except for the two isolates (ST752 and ST4980) which demonstrated reduced susceptibility to ciprofloxacin with MICs of 0.125 and 0.25 mg/L, respectively. These detections of non-wild-type resistance to fluoroquinolones were unexpected since the use of this class has never been permitted in food animals in Australia. Nevertheless, fluoroquinolone resistance has also recently been described in four *E*. *coli* (ST10) isolates from healthy pigs at slaughter [12] as well as a single *E*. *coli* isolate (ST10) from a diseased pig [11]. ST752 is a relatively broad host range sequence type that has been isolated globally from humans, animals and the environment, while ST4980 has been isolated from poultry farms in Europe and North America [33–36]. Recent studies on livestock *E*. *coli* in Australia have revealed that reverse zoonotic transmission and/or migratory avian species may have a role in the introduction of human-associated, CIA resistant *E*. *coli* in Australian livestock systems [11, 12, 26, 37]. Importation of live poultry into Australia is an unlikely source of these organisms because it occurs infrequently and is confined to fertile eggs from flocks with known disease status, with eggs having undergone treatment to remove microbiological contaminants [38]. In addition, there are strictly-enforced biosecurity measures at the national border to exclude illegal entry of live poultry and poultry products [38]. Based on the above, and given the chromosomal resistance mechanisms such as the QRDR mutations detected here do not transfer horizontally amongst bacteria, it is most likely that this resistance was introduced into Australian poultry rather than evolving under quinolone selection pressure.

Although resistance to highest priority CIAs such as ceftiofur, ciprofloxacin and colistin were not detected among *Salmonella* isolates, a small proportion (11.3%) of isolates demonstrated reduced susceptibility to cefoxitin. DNA sequence analysis revealed that none of these isolates carried any of the known beta-lactam resistance genes imparting resistance to cefoxitin. It is quite likely that the reduced susceptibility of these *Salmonella* isolates to cefoxitin arises from the variable performance of phenotypic assays resulting in a phenomenon known as MIC shift, the occurrence of which has been shown using *E*. *coli* to result in sub-optimal assay performance for particular drugs including cefoxitin [39].

The intention of this study was to produce data for interpretation at the national-level. While it is possible to compare enterprises in the proportion of isolates resistant to each drug this is not statistically valid owing to the extent of “multiple-comparisons” that promote the occurrence of Type 1 statistical errors (declaration of false-positive associations). Technology involving robotics and high throughput liquid handlers currently being developed will provide opportunities for future studies based on high-volume processing of isolates to be undertaken in a manner that allows larger numbers of isolates per establishment to be assessed in combination with a more sophisticated data analysis.

Since a national review of AMR in animals was initiated in 1996 [40], there has been substantial scrutiny of all aspects of antimicrobial use in food-producing animals in Australia. This has encouraged interest in antimicrobial stewardship at the flock level supported at the national level by regulations effectively preventing the use of highest priority critically important antimicrobials in poultry (notably fluoroquinolones, ESCs and polymixins) [30]. There has also been widespread use of efficacious vaccines for major poultry pathogens such as *Pasteurella* and *Mycoplasma* spp., and an overall enhancement of biosecurity measures in response to a number of costly outbreaks of exotic viral diseases. Notwithstanding the progress that has been made, the results of which are demonstrated in this paper, there is a need for industry and government to proactively monitor AMR and antimicrobial stewardship to advise policy on management of antimicrobials in the livestock sector for long-term protection of both animal and human health.
