# Correction: *Escherichia coli* and *Salmonella* spp. isolated from Australian meat chickens remain susceptible to critically important antimicrobial agents

**DOI:** 10.1371/journal.pone.0227383

**Published:** 2020-01-13

**Authors:** Sam Abraham, Mark O’Dea, Shafi Sahibzada, Kylie Hewson, Anthony Pavic, Tania Veltman, Rebecca Abraham, Taha Harris, Darren J. Trott, David Jordan

There are errors in Tables 2 and 4. The vertical bars representing ECOFFs are not visible in Tables 2 and 4.

There is an additional error in the title of Table 4. The value for number of Salmonella (n) is 53, not 206. Please see the correct tables here.

**Table 2:** Distribution (percent of isolates) of minimum inhibitory concentrations (mg/L) for commensal *Escherichia coli* (n = 206) isolated from Australian meat chickens at slaughter.

Antimicrobial	Class	0.016	0.03	0.06	0.13	0.25	0.5	1	2	4	8	16	32	64	128	256	Percent non-wildtype (95% CI)
Amoxicillin-clavulanate	bla-i							5.3	39.3	42.7	12.6						0 (0–1.8)
Ampicillin	bla							19.9	44.2	21.4	0.5			14.1			14.1 (9.6–19.0)
Cefoxitin	c2g								4.9	70.4	24.3	0.5					0.5 (0–2.7)
Ceftiofur *	c3g				1.5	45.1	52.4	1									0 (0–1.8)
Ceftriaxone *	c3g					100											0 (0–1.8)
Chloramphenicol	phe								4.4	43.7	51.9						0 (0–1.8)
Ciprofloxacin *	qui	95.1	3.9		0.5	0.5											1 (0.1–3.5)
Colistin *	pol				21.8	73.3	3.4	1.5									0 (0–1.8)
Florfenicol	phe									9.7	76.2	14.1					0 (0–1.8)
Gentamicin	ami					5.8	79.1	15									0 (0–1.8)
Streptomycin	ami								1	50	36.9	2.4	4.9	2.4	2.4		9.7 (6–14.6)
Tetracycline	tet									80.6				19.4			19.4 (14.2–25.5)
Trimethoprim/sulf	fpi				87.9	1.5	1.5	0.5			8.7						8.7 (5.3–13.5)

The shaded areas indicate the range of dilutions tested for each antimicrobial. ECOFF values are shown with vertical bars.

ami = aminoglycosides, bla = beta lactams, bla-i = beta lactams/inhibitor, c2g = 2^nd^ generation cephalosporins, c3g = 3^st^ generation cephalosporin, fpi = folate pathway inhibitors, phe = phenicols, pol = polymixins, qui = quinolones, tet = tetracycline

*—Critically important antimicrobial

**Table 4:** Distribution (percent of isolates) of minimum inhibitory concentrations (mg/L) for *Salmonella* spp (n = 53) isolated from Australian meat chickens at slaughter.

Antimicrobial	Class	0.016	0.03	0.06	0.13	0.25	0.5	1	2	4	8	16	32	64	128	256	Percent non-wildtype (95% CI)
Amoxicillin-clavulanate	bla-i							77.4	17	1.9	1.9	1.9					3.8 (0.5–13)
Ampicillin	bla							67.9	28.3					3.8			3.8 (0.5–13)
Cefoxitin	c2g								34	41.5	13.2	11.3					11.3 (4.3–23)
Ceftiofur *	c3g					1.9	18.9	64.2	15.1								0 (0–6.7)
Ceftriaxone *	c3g					100											0 (0–6.7)
Chloramphenicol	phe									45.3	54.7						0 (0–6.7)
Ciprofloxacin *	qui	49.1	50.9														0 (0–6.7)
Colistin *	pol					9.4	60.4	30.2									0 (0–6.7)
Florfenicol	phe									24.5	71.7	3.8					0 (0–6.7)
Gentamicin	ami					66	34										0 (0–6.7)
Streptomycin	ami									20.8	60.4	17			1.9		1.9 (0–10.1)
Tetracycline	tet									100							0 (0–6.7)
Trimethoprim/sulfa	fpi				96.2	1.9					1.9						1.9 (0–10.1)

The shaded areas indicate the range of dilutions tested for each antimicrobial. ECOFF values are shown with vertical bars.

ami = aminoglycosides, bla = beta lactams, bla-i = beta lactams/inhibitor, c2g = 2^nd^ generation cephalosporins, c3g = 3^st^ generation cephalosporin, fpi = folate pathway inhibitors, phe = phenicols, pol = polymixins, qui = quinolones, tet = tetracycline

*—Critically important antimicrobial
